# Effects of circulating inflammatory proteins on osteoporosis and fractures: evidence from genetic correlation and Mendelian randomization study

**DOI:** 10.3389/fendo.2024.1386556

**Published:** 2024-05-01

**Authors:** Qingcong Zheng, Du Wang, Rongjie Lin, Zhechen Li, Yuchao Chen, Rongsheng Chen, Chunfu Zheng, Weihong Xu

**Affiliations:** ^1^ Department of Spinal Surgery, The First Affiliated Hospital of Fujian Medical University, Fuzhou, China; ^2^ Arthritis Clinical and Research Center, Peking University People’s Hospital, Beijing, China; ^3^ Department of Orthopedic Surgery, Fujian Medical University Union Hospital, Fuzhou, China; ^4^ Department of Paediatrics, Fujian Provincial Hospital South Branch, Fuzhou, China; ^5^ Department of Microbiology, Immunology and Infectious Diseases, University of Calgary, Calgary, AB, Canada

**Keywords:** osteoporosis, fracture, bone homeostasis, circulating inflammatory proteins, Mendelian randomization

## Abstract

**Objective:**

There is a controversy in studies of circulating inflammatory proteins (CIPs) in association with osteoporosis (OP) and fractures, and it is unclear if these two conditions are causally related. This study used MR analyses to investigate the causal associations between 91 CIPs and OP and 9 types of fractures.

**Methods:**

Genetic variants data for CIPs, OP, and fractures were obtained from the publicly available genome-wide association studies (GWAS) database. We used inverse variance weighted (IVW) as the primary analysis, pleiotropy, and heterogeneity tests to analyze the validity and robustness of causality and reverse MR analysis to test for reverse causality.

**Results:**

The IVW results with Bonferroni correction indicated that CXCL11 (OR = 1.2049; 95% CI: 1.0308-1.4083; *P* = 0.0192) can increase the risk of OP; IL-4 (OR = 1.2877; 95% CI: 1.1003-1.5070; *P* = 0.0016), IL-7 (OR = 1.2572; 95% CI: 1.0401-1.5196; *P* = 0.0180), IL-15RA (OR = 1.1346; 95% CI: 1.0163-1.2668; *P* = 0.0246), IL-17C (OR = 1.1353; 95% CI: 1.0272-1.2547; *P* = 0.0129), CXCL10 (OR = 1.2479; 95% CI: 1.0832-1.4377; *P *= 0.0022), eotaxin/CCL11 (OR = 1.1552; 95% CI: 1.0525-1.2678; *P* = 0.0024), and FGF23 (OR = 1.9437; 95% CI: 1.1875-3.1816; *P* = 0.0082) can increase the risk of fractures; whereas IL-10RB (OR = 0.9006; 95% CI: 0.8335-0.9730; *P* = 0.0080), CCL4 (OR = 0.9101; 95% CI: 0.8385-0.9878; *P* = 0.0242), MCP-3/CCL7 (OR = 0.8579; 95% CI: 0.7506-0.9806; *P* = 0.0246), IFN-γ [shoulder and upper arm (OR = 0.7832; 95% CI: 0.6605-0.9287; *P* = 0.0049); rib(s), sternum and thoracic spine (OR = 0.7228; 95% CI: 0.5681-0.9197; *P* = 0.0083)], β-NGF (OR = 0.8384; 95% CI: 0.7473-0.9407; *P* = 0.0027), and SIRT2 (OR = 0.5167; 95% CI: 0.3296-0.8100; *P* = 0.0040) can decrease fractures risk.

**Conclusion:**

Mendelian randomization (MR) analyses indicated the causal associations between multiple genetically predicted CIPs and the risk of OP and fractures.

## 1 Introduction

Osteoporosis is a common systemic metabolic bone disease characterized by an imbalance in bone homeostasis, microstructural disruption of bone tissue, decreased bone mineral density (BMD), and an increased risk of fracture (1), with fracture being the most serious outcome of OP (2). Studies have shown that the global prevalence of OP was 18.3% (3) and was positively correlated with age, with worldwide prevalence rates of OP corresponding to 11.4%/24.8%/37.6%/40.8% in the 50-59/60-69/70-79/89-89 age groups (4). In addition, the total number of fragility fractures in the EU6 was projected to increase to 3.3 million by 2030 (a rise of 23%), with a 27% increase in annual fracture-related costs (5). The trend of population aging is contributing to the increasing number of OPs and fractures, which is a major global public health problem and imposes a heavy health and economic burden on individuals and societies (6).

The term “osteoimmunology” was coined in 2000 and has been a hot topic of research in recent years (7), with CIPs being the key bridge between the interconnectedness of the immune system and the skeletal system (8). OP is considered an inflammatory bone anomaly. However, the role of CIPs in OP and fractures is complex and not yet fully unraveled (9). Inflammation and inflammatory proteins not only induce OP by affecting bone strength and quality (10) but also contribute to increased fracture risk by inhibiting bone formation, promoting bone loss, and impairing bone regeneration (11, 12). However, inflammation and inflammatory proteins can also inhibit OP by promoting osteoblast (OB) differentiation, further inducing osteogenesis through multiple signaling pathways (13), and being key factors in promoting early fracture healing and bone regeneration (14). Studies on the association between CIPs and OP and fractures have been both mutually supportive and contradictory, with bias due to confounding factors, environmental factors, and reverse causation being one of the main reasons for the conflicting nature of these studies.

Exploring the causal associations between CIPs and the risk of OP and fractures in terms of genetic factors has not yet been clearly reported. MR is an epidemiological research method that analyses summary-level data from GWAS by means of reliable instrumental variables (IVs). Due to the high degree of randomness of genetic variation and the fact that alleles are not affected by the environmental factors of the disease, MR can further reliably infer causality between exposures and outcomes by greatly reducing bias due to confounding factors, environmental factors, and reverse causality (15). This study used a bidirectional Mendelian randomization method to investigate whether there are genetically predicted causal associations between CIPs and the risk of OP and fractures.

## 2 Materials and methods

### 2.1 Study design

Our study was done under the guidance of “strengthening the reporting of observational studies in epidemiology using Mendelian randomization (STROBE-MR)” (16). The data we used were obtained from publicly available GWAS database, and ethics committees have approved these original studies. Therefore, no ethical approval is required to cite these public datasets. SNPs used as valid IVs in MR analyses must satisfy the following three key assumptions. (i) The relevance assumption, the IVs must be directly and strongly related to the exposure; (ii) the independence assumption, the IVs must be unrelated to any confounding factors; (iii) the exclusion restriction assumption: the IVs can only affect outcomes through exposures (no directional pleiotropy) (**Supplementary Figure S1**).

### 2.2 GWAS data sources

In the present study, summary-level statistics for both exposure and outcome were derived from European ancestry, which could reduce bias due to race-related confounders. *Exposures.* We obtained 91 CIPs datasets (accession numbers from GCST90274758 to GCST90274848) from GWAS (https://www.ebi.ac.uk/gwas/home) (17). *Outcomes.* We searched the Integrative Epidemiology Unit (IEU, https://gwas.mrcieu.ac.uk/) for The summary data of OP (finn-b-M13_OSTEOPOROSIS) and 9 types of fractures (3 major sites each in the upper limb, lower limb, and midshaft bones), including fracture of shoulder and upper arm (finn-b-ST19_FRACT_SHOUL_UPPER_ARM), fracture of forearm (finn-b-ST19_FRACT_FOREA), fracture at wrist and hand level (finn-b-ST19_FRACT_WRIST_HAND_LEVEL), fracture of femur (finn-b-ST19_FRACT_FEMUR), fracture of lower leg, including ankle (finn-b-ST19_FRACT_LOWER_LEG_INCLU_ANKLE), Fracture of foot, except ankle (finn-b-ST19_FRACT_FOOT_ANKLE), fracture of neck (finn-b-ST19_FRACT_NECK), fracture of rib(s), sternum and thoracic spine (finn-b-ST19_FRACT_RIBS_STERNUM_THORACIC_SPINE), and fracture of lumbar spine and pelvis (finn-b-ST19_FRACT_LUMBAR_SPINE_PELVIS). After comparing the sources of participants in the 91 datasets from the CIPs with the 10 datasets from the skeletal system, we considered the samples of the GWAS data for exposures and outcomes to be independent of each other as a way of reducing bias due to overlapping data sources. We summarise the details of these data in **Supplementary Table S1**.

### 2.3 Selection of instrumental variables

We filtered out SNPs in the 91 CIPs dataset by setting a significance threshold of “*5E-06*”. These SNPs were analyzed by the “*clump_data*” function for linkage disequilibrium at “*r^2^ < 0.001, 10000 kb*” in order to exclude mutual linkage SNPs and to discard non-biallelic SNPs to ensure independence among IVs for each exposure. We assessed the strength of association between the screened SNPs and exposure using F-statistic as a way to avoid bias caused by weak IVs. The F-statistic is calculated as *F = (β^2^/standard error^2^)*, when F > 10 for SNPs shows that it is strongly effective IVs (18). When the effect alleles for the SNPs’ effects in the exposure and outcome were different, the summary set might generate errors. We used the “*harmonise_data*” function to test the causal direction of the selected SNPs in the exposure and outcome, eliminated the palindromic alleles, and finally chose the SNPs with the result of “*TRUE*” as the effective IVs.

### 2.4 Two-sample Mendelian randomization analysis

We performed the TSMR analysis with the “*TwoSampleMR*” package of the R version 4.2.3 software. Forward MR was analyzed with CIPs as exposures and OP and fractures as outcomes. The purpose of reverse MR analyses by interchanging exposures and outcomes was to exclude bias due to reverse causality. First, we used five methods, MR Egger, weighted median, random effects IVW, simple mode, and weighted mode, to assess the causal associations between exposures and outcomes, with IVW being the most reliable. With “*IVW P-value < 0.05*”, we applied the Bonferroni correction to the results using the “*p.adjust*” function in the R software and recorded the exposure factors of *P_adjust_* < 0.05 and set *P_adjust_* < 0.1 if the screening was fruitless. The results of IVs, all-MR Egger, and all-IVW were visualized using a forest plot. Second, the heterogeneity test. We used Cochran’s Q-statistic for heterogeneity analysis of SNPs in IVW and MR-Egger analyses in order to assess the robustness of IVs when *P-value* > 0.05 indicated that there was no significant heterogeneity in the results (19). The test results for heterogeneity were visualized by funnel plots of the IVs. Third, the pleiotropy test. Pleiotropy refers to the fact that some IVs affect outcomes through confounding factors other than exposures, which would seriously undermine the reliability of the causal associations between exposures and outcomes. We tested for outliers by MR-PRESSO (global test *P-value* < 0.05) and reassessed the causal association between exposure and outcome after excluding outliers (20). At the same time, we used the “*MR_pleiotropy_test*” function for effect estimation and bias detection of MR-Egger intercept, and when the *P-value >*0.05 showed no evidence of significant pleiotropy (21). Leave-one-out analysis was performed by sequentially removing single SNPs and then rerunning the IVW analysis to assess the effect of the remaining SNPs on the outcome, with the aim of discovering whether there were any single SNPs driving causality. Finally, we used the mRnd website (https://shiny.cnsgenomics.com/mRnd/) to assess the statistical power of the MR analysis. α (type-I error rate) was set to 0.05, and R^2^ (proportion of variance explained for the association between the SNP or allele score and the exposure variable) was calculated as *2×eaf×(1-eaf)×β^2^* (22, 23).

## 3 Results

### 3.1 Forward MR

**Figure 1:**
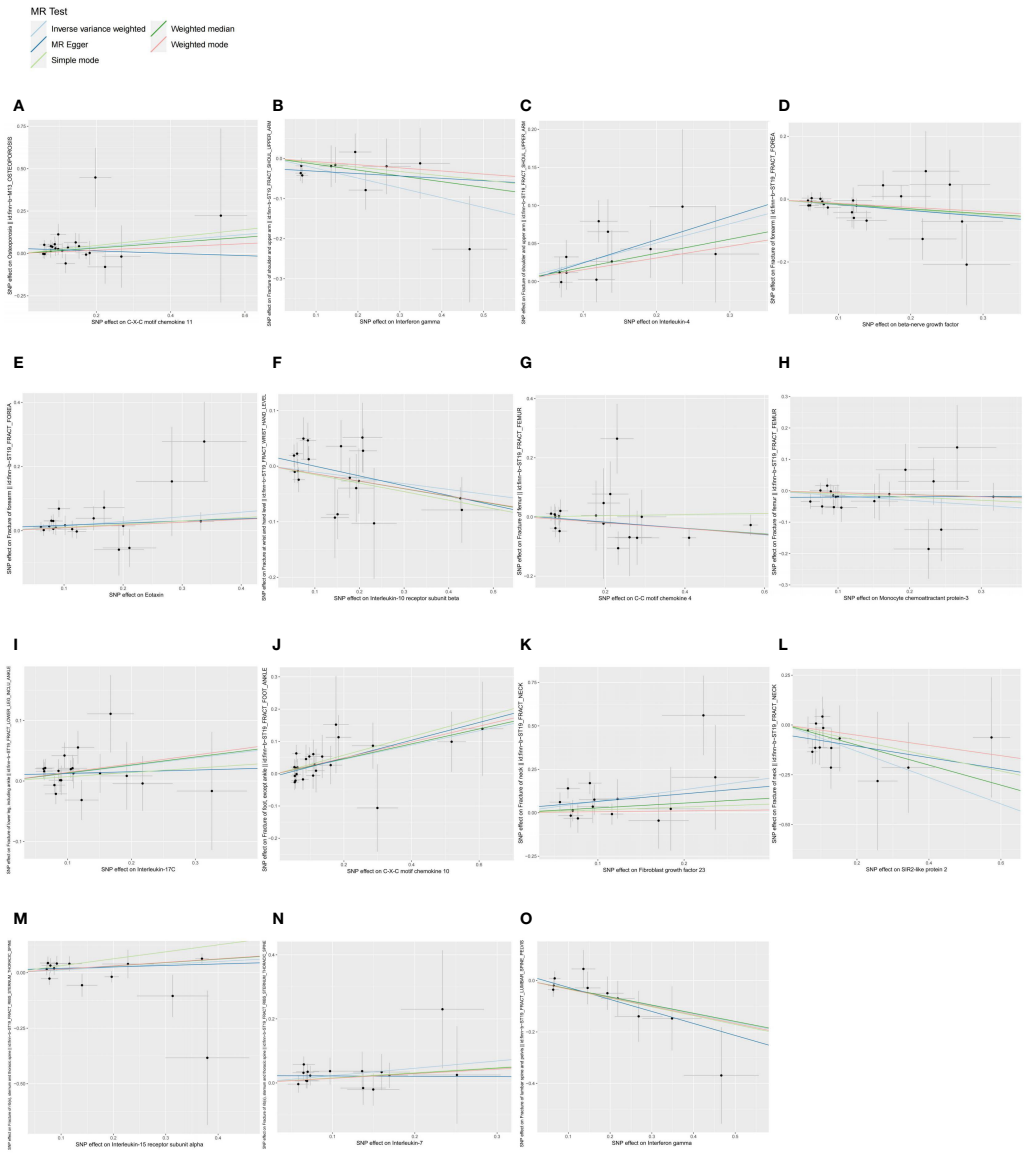
Scatter plots of causal associations between exposures (CIPs) and outcomes (OP, fractures). **(A)** Scatter plot between CXCL11 and OP; **(B)** Scatter plot between IFN-γ and fracture of shoulder and upper arm; **(C)** Scatter plot between IL-4 and fracture of shoulder and upper arm; **(D)** Scatter plot between β-NGF and fracture of forearm; **(E)** Scatter plot between eotaxin and fracture of forearm; **(F)** Scatter plot between IL-10RB and fracture at wrist and hand level; **(G)** Scatter plot between CCL4 and fracture of femur; **(H)** Scatter plot between MCP-3 and fracture of femur; **(I)** Scatter plot between IL-17C and fracture of lower leg (including ankle); **(J)** Scatter plot between CXCL10 and fracture of foot (except ankle); **(K)** Scatter plot between FGF23 and fracture of neck; **(L)** Scatter plot between SIRT2 and fracture of neck; **(M)** Scatter plot between IL-15RA and fracture of rib(s), sternum and thoracic spine; **(N)** Scatter plot between IL-7 and fracture of rib(s), sternum and thoracic spine; **(O)** Scatter plot between IFN-γ and fracture of lumbar spine and pelvis.

**Figure 2:**
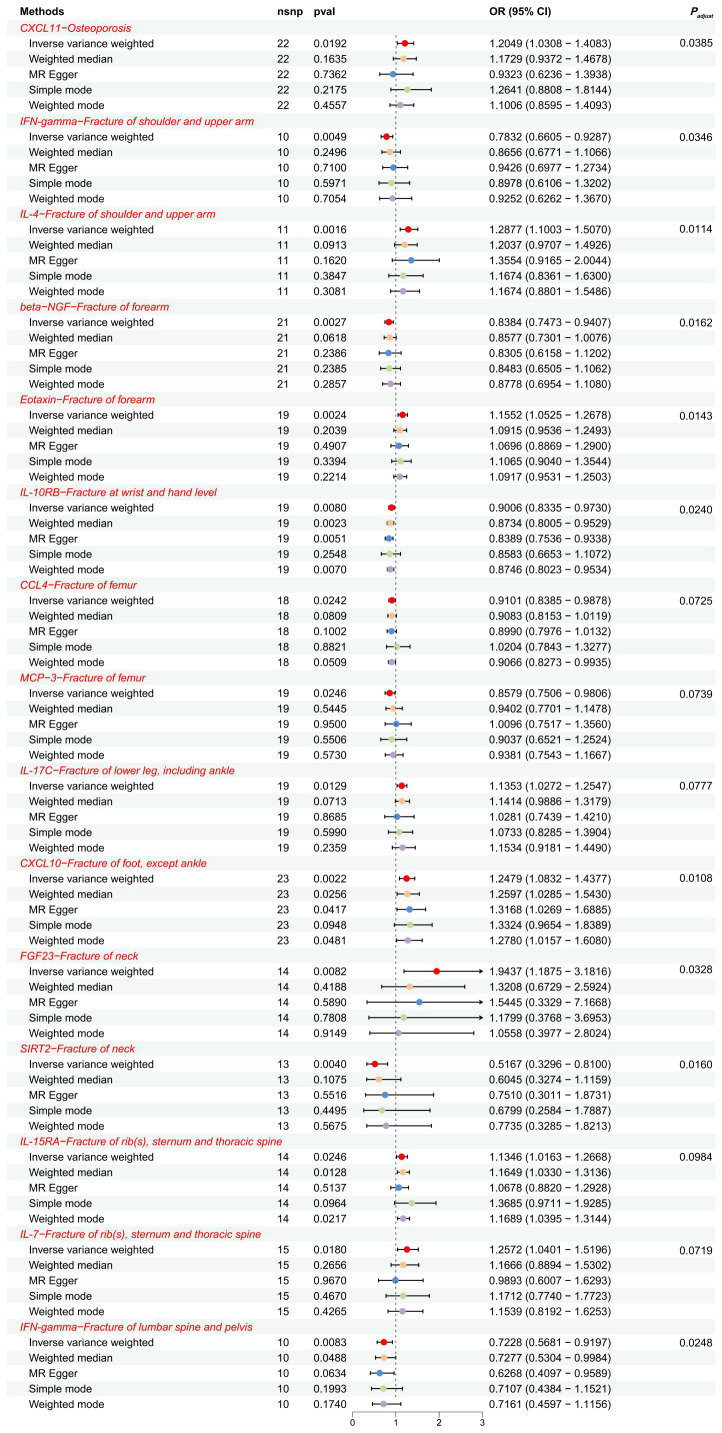
The forest plot showed the causal associations between exposures (CIPs) and outcomes (OP, fractures) in the forward MR analysis. SNP, single-nucleotide polymorphism; OR, odds ratio; 95% CI, 95% confidence interval.

Forward MR and the sensitivity analysis results are in **Supplementary Table S2**, and detailed information on IVs and the results of power analysis are in **Supplementary Table S3**, where SNPs as IVs were all strong instrumental variables (F-statistic > 10). IVW analysis (**Figure 1**) showed that CXCL11 (OR = 1.2049; 95% CI: 1.0308-1.4083; *P* = 0.0192; *P_adjust_* = 0.0385) can increase OP risk. For fracture of the shoulder and upper arm, IFN-γ (OR = 0.7832; 95% CI: 0.6605-0.9287; *P* = 0.0049; *P_adjust_* = 0.0346) can reduce its risk, whereas IL-4 (OR = 1.2877; 95% CI: 1.1003-1.5070; *P* = 0.0016; *P_adjust_* = 0.0114) can increase its risk. For fracture of forearm, β-NGF (OR = 0.8384; 95% CI: 0.7473-0.9407; *P* = 0.0027; *P_adjust_* = 0.0162) can reduce its risk, while Eotaxin/CCL11 (OR = 1.1552; 95% CI: 1.0525-1.2678; *P* = 0.0024; *P_adjust_* = 0.0143) can increase its risk. IL-10RB (OR = 0.9006; 95% CI: 0.8335-0.9730; *P* = 0.0080; *P_adjust_* = 0.0240) can reduce the risk of fracture at the wrist and hand level. CCL4 (OR = 0.9101; 95% CI: 0.8385-0.9878; *P* = 0.0242; *P_adjust_* = 0.0725) and MCP-3/CCL7 (OR = 0.8579; 95% CI: 0.7506-0.9806; *P* = 0.0246; *P_adjust_* = 0.0739) can reduce the risk of fracture of the femur. IL-17C (OR = 1.1353; 95% CI: 1.0272-1.2547; *P* = 0.0129; *P_adjust_* = 0.0777) can increase the risk of fracture of the lower leg (including the ankle). CXCL10 (OR = 1.2479; 95% CI: 1.0832-1.4377; *P* = 0.0022; *P_adjust_* = 0.0108) can increase the risk of fracture of the foot (except ankle). For fracture of the neck, FGF23 (OR = 1.9437; 95% CI: 1.1875-3.1816; *P* = 0.0082; *P_adjust_* = 0.0328) can increase its risk, while SIRT2 (OR = 0.5167; 95% CI: 0.3296-0.8100; *P* = 0.0040; *P_adjust_* = 0.0160) can decrease its risk. IL-15RA (OR = 1.1346; 95% CI: 1.0163-1.2668; *P* = 0.0246; *P_adjust_* = 0.0984) and IL-7 (OR = 1.2572; 95% CI: 1.0401-1.5196; *P* = 0.0180; *P_adjust_* = 0.0719) can increase the risk of fracture of rib(s), sternum and thoracic spine. IFN-γ (OR = 0.7228; 95% CI: 0.5681-0.9197; *P* = 0.0083; *P_adjust_* = 0.0248) can reduce the risk of fracture of lumbar spine and pelvis. We presented the above results in a summarised forest plot (**Figure 2**) and used a forest plot to visualize the results for IVs, all-MR Egger, and all-IVW (**Supplementary Figure S2**). In the sensitivity analyses, the *P-value* for the pleiotropy tests (MR-egger intercept test and MR-PRESSO global test) and the heterogeneity test (Cochran’s Q-statistic) for these 15 causal associations were all greater than 0.05. SNPs were symmetrically distributed in the funnel plot of IVW (**Supplementary Figure S3**). No obvious single SNP was found to have an influence on the association in the leave-one-out analysis plot (**Supplementary Figure S4**). In conclusion, sensitivity analyses showed no significant pleiotropy or heterogeneity in these results, implying the high validity and robustness of the MR analyses.

### 3.2 Reverse MR

**Figure 3:**
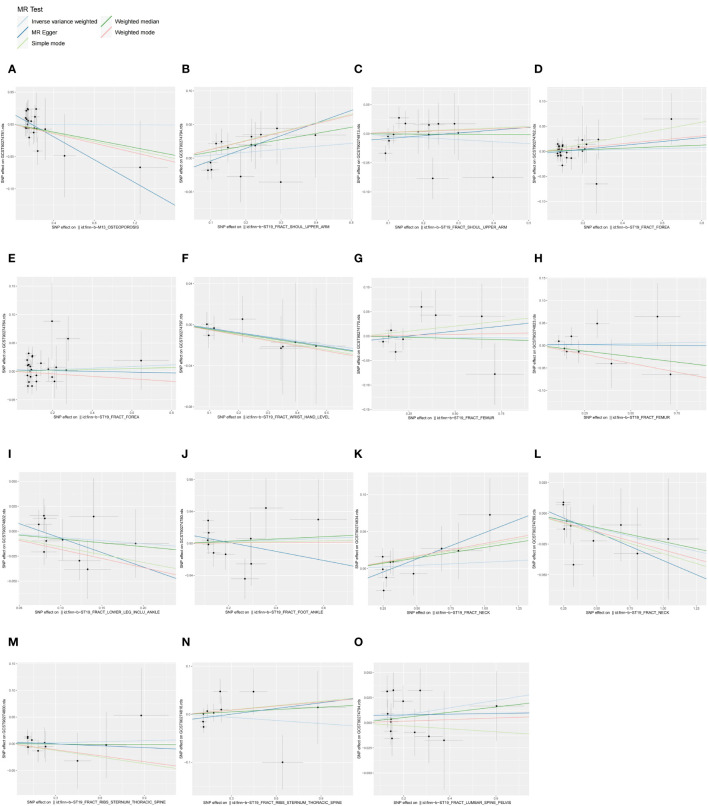
Scatter plots of causal associations between exposures (OP, fractures) and outcomes (CIPs). **(A)** Scatter plot between OP and CXCL11; **(B)** Scatter plot between fracture of shoulder and upper arm and IFN-γ; **(C)** Scatter plot between fracture of shoulder and upper arm and IL-4; **(D)** Scatter plot between fracture of forearm and β-NGF; **(E)** Scatter plot between fracture of forearm and eotaxin; **(F)** Scatter plot between fracture at wrist and hand level and IL-10RB; **(G)** Scatter plot between fracture of femur and CCL4; **(H)** Scatter plot between fracture of femur and MCP-3; **(I)** Scatter plot between fracture of lower leg (including ankle) and IL-17C; **(J)** Scatter plot between fracture of foot (except ankle) and CXCL10; **(K)** Scatter plot between fracture of neck and FGF23; **(L)** Scatter plot between fracture of neck and SIRT2; **(M)** Scatter plot between fracture of rib(s), sternum and thoracic spine and IL-15RA; **(N)** Scatter plot between fracture of rib(s), sternum and thoracic spine and IL-7; **(O)** Scatter plot between fracture of lumbar spine and pelvis and IFN-γ.

**Figure 4:**
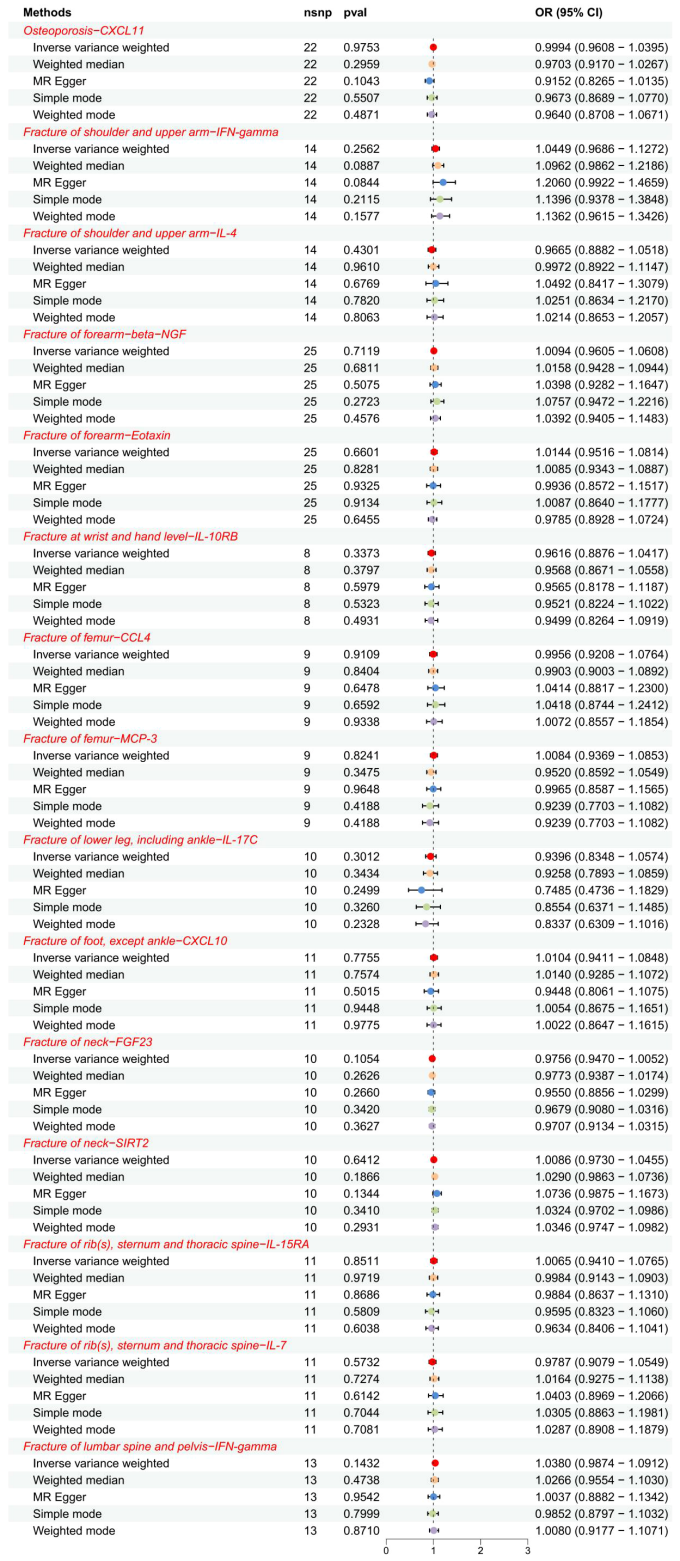
The forest plot showed the causal associations between exposures (OP, fractures) and outcomes (CIPs) in the reverse MR analysis. SNP, single-nucleotide polymorphism; OR, odds ratio; 95% CI, 95% confidence interval.

We similarly screened IVs for OP and fractures datasets on the basis of setting a significance threshold of “5E-06”. Reverse MR and the sensitivity analysis results are in **Supplementary Table S4**, and detailed information on IVs is in **Supplementary Table S5**, where SNPs as IVs were all strong instrumental variables (F-statistic > 10). IVW analysis showed (**Figure 3**) that there was no evidence to support the causal associations of OP with CXCL11(*P* = 0.9753), fracture of the shoulder and upper arm with IFN-γ (*P *= 0.2562) and IL-4 (*P *= 0.4301), fracture of the forearm with β-NGF (*P* = 0.7119) and eotaxin/CCL11 (*P* = 0.6601), fracture at wrist and hand level with IL-10RB (*P* = 0.3373), fracture of femur with CCL4 (*P* = 0.9109) and MCP-3/CCL7 (*P* = 0.8241), fracture of lower leg (including ankle) with IL-17C(*P *= 0.3012), fracture of foot (except ankle) with CXCL10 (*P* = 0.7755), fracture of rib (s), sternum and thoracic spine with IL-15RA (*P* = 0.8511) and IL-7 (*P* = 0.5732), fracture of lumbar spine and pelvis with IFN-γ (*P* = 0.1432). Since the fracture of neck dataset was screened for too few SNPs at a significance threshold of “*5E-06*”, MR analysis was performed using a significance threshold of “*1E-05*” and found no evidence to support a causal association between the levels of FGF23 (*P* = 0.1054) or SIRT2 (*P* = 0.6412) in the fracture of neck dataset. In MR analysis of the fracture of the forearm on eotaxin/CCL11, although Cochran’s Q *P-value* < 0.05 for IVW indicated heterogeneity, heterogeneity was considered acceptable in the present study in the case of using random-effects IVW (24). In the above reverse, MR, the *P-values* of pleiotropy tests (MR-egger intercept test and MR-PRESSO global test) and heterogeneity test (Cochran’s Q-statistic) were all greater than 0.05, indicating no significant pleiotropy and heterogeneity, and high validity and robustness of the MR analyses. We presented the results of the reverse MR analyses in a summarised forest plot (**Figure 4**) and attached forest plots (**Supplementary Figure S5**), funnel plots (**Supplementary Figure S6**), and leave-one-out analysis plots (**Supplementary Figure S7**) to the **Supplementary Material**. In conclusion, the reverse MR results indicate that there is no obvious bias caused by reverse causality in the forward MR.

## 4 Discussion

Bone homeostasis is maintained by the balance between the OB of bone formation and the osteoclast (OC) of bone resorption, as well as by intraosseous vascular homeostasis. OP and fractures are inextricably linked to an imbalance in bone homeostasis, and CIPs play a critical and complex role in the regulation of bone homeostasis. To our knowledge, this is the first MR analysis to explore the causal associations between 91 CIPs and the risk of OP and 9 types of fractures. In analyzing the current GWAS datasets cited in this study, we found that CXCL11 can increase the risk of OP; IL-4, IL-7, IL-15RA, IL-17C, CXCL10, eotaxin/CCL11, FGF23 can increase the risk of fractures, whereas IL-10RB, CCL4, MCP-3/CCL7, IFN-γ, β-NGF, SIRT2 can decrease the risk of fractures, and there is no reverse causality of the above results.

### 4.1 Association of interleukins with osteoporosis and fractures

Studies have shown that IL-4 can reduce mature OC activity to further inhibit bone resorption through NF-κB and Ca^2+^ signaling pathways (25). Other studies have reported that IL-4 readily induces M2 macrophages to differentiate into OC (26) and that the IL-4/IL-4R pathway promotes the proliferation of preosteoclast cells to provide a large number of “seeds” for OC (27). Controversy exists regarding the role of IL-4 in the skeletal system, and we found that genetically predicted IL-4 can increase fracture risk. OC is the only bone-resorbing cell in the body, and osteoclastogenic cytokines, including IL-7, IL-15, and IL-17, and anti-osteoclastogenic cytokines, including IL-10, form a key signaling network that regulates OC proliferation and differentiation (28). IL-7 not only induces T cells to secrete receptor activator of nuclear factor kappa-B ligand (RANKL) and TNF-α to enhance OC proliferation and thereby induce bone loss (29) but also induces OC production by the STAT5 pathway, which is independent of the RANKL pathway (30). Controversially, some studies have found that IL-7 is a direct inhibitor of OC generation *in vitro* (31). IL-15 not only increases the number of OC by stimulating the differentiation of OC progenitor into OC precursor (32) but also synergizes with the RANKL pathway (33) and the phospholipase D1 (PLD1) pathway (34) to promote OC production, further contributing to bone destruction and bone loss. Downregulation of IL-15 levels inhibits the RANKL-RANK- osteoprotegerin (OPG) axis and reduces the risk of femoral head necrosis (35). Controversially, IL-15 is also essential in OB function as well as bone mineralization (36). As an inducer of RANKL, IL-17 not only stimulates OC proliferation, leading to bone erosion (37) but also enhances OC activity, thereby causing bone destruction (38). Controversially, some studies have reported that IL-17 promotes OB secretion of granulocyte-macrophage colony-stimulating factor (GM-CSF) to inhibit differentiation of preosteoclast cells (39). IL-10, an osteoblast factor, can hinder OC differentiation and maturation by inhibiting RANK and RANKL and promoting OPG expression, and high levels of IL-10 can inhibit OC activity (40). Other studies have reported that low concentrations of IL-10 can induce bone formation through p38 MAPK signaling, but high concentrations of IL-10 cause bone damage (41). In conclusion, the roles of interleukins in the skeletal system and bone homeostasis are complex and varied, and many studies are controversial or even have opposite conclusions, with bias due to confounding factors, environmental factors, and reverse causality being one of the main reasons for the conflicting nature of these studies. We have shown by MR analysis that IL-4, IL-7, IL-15RA, and IL-17C promote fractures, whereas IL-10RB can decrease fracture risk, which provides genetic evidence for research in this area.

### 4.2 Association of chemokines with osteoporosis and fractures

Chemokines comprise two major families (CXC, CC) that are key signals for the migration and localization of circulating cells into various tissues and play an important role in bone metabolism. CXCL10 enhances the homing and differentiation of circulating osteoclast progenitor cells and stimulates OC production. Thus, it is considered an osteoclastogenic factor and has osteoclastogenic effects (42). The C5a/C5aR1 axis is strongly associated with fractures, and CXCL10 production is a key effector outcome of this pathway (43). In addition, bone erosion was prevented by vitamin D supplementation that inhibited the CXCL10 pathway (44). It has also been shown that there is no significant correlation between CXCL10 and hip fracture risk (45). Our MR analysis revealed that genetically predicted CXCL10 can promote fracture occurrence. CXCL11 inhibits angiogenesis, and impaired angiogenesis is a key pathophysiological microenvironmental condition in OP (46). Some studies have suggested that CXCL11 can inhibit the differentiation of monocytes to OC (47). However, other studies have suggested that CXCL11 has no significant role in OC differentiation (48). Studies on the association between CXCL11 and OP are scarce and controversial, and the present study demonstrates that genetically predicted CXCL11 promotes the progression of OP.

Studies have concluded that CCL4 can promote OC invasion and induce bone resorption disease through OC differentiation gene expression profiling (49). Controversially, other studies have suggested that although CCL4 promotes the viability and migratory capacity of preosteoclast cells, it is not required for OC differentiation and is not directly involved in OC generation; furthermore, CCL4 improves function in the OB ecological niche by recruiting progenitor cells and maintaining viability (50). Studies have shown that MCP-3/CCL7 can exacerbate osteolysis by promoting RANKL generation and bone-resorptive OC recruitment (51). Controversially, Other studies have shown that CCL7 can induce OB homing to the fracture site to participate in repair (52). And CCL7 has a role in promoting angiogenesis (53). It has been reported that eotaxin/CCL11 can promote the invasion of preosteoclast cells and lead to bone resorption, so it is considered a novel inflammatory bone resorption factor (54). In conclusion, CCL4 and CCL7 are more similar to a regulator of bone homeostasis. However, there are fewer reports on the relevance of CCL4/CCL7/CCL11 in bone homeostasis, which is a direction that deserves to be explored in depth. Our MR analyses indicated that genetically predicted CCL4 and CCL7 can decrease fracture risk, whereas CCL11 can increase fracture risk.

### 4.3 Association of other CIPs with osteoporosis and fractures

As an osteoblast factor, IFN-γ plays an important regulatory role in osteoimmunology. IFN-γ not only promotes bone mineralization by stimulating OB differentiation through induction of the runt-related transcription factor 2 (RUNX2)/osterix (OSX) pathway but also reduces bone loss by inhibiting OC activity (55, 56). Controversially, other studies have reported that IFN-γ can promote apoptosis in OB synergistically through the activation of caspases, and it can also promote OC production under specific conditions causing bone destruction (57, 58). OB secretes NGF in response to mechanical loading and can be innervated via the NGF-tropomyosin receptor kinase A (TrkA) signaling pathway to further drive bone formation and maintain bone homeostasis *in vivo* (59, 60). NGF deficiency will reduce the migration of osteogenic precursors to the injury site, causing delayed bone healing (61). SIRT2 is the most richly expressed factor of the class III family of histone deacetylases (HDAC-III) in human bone tissue, which can prevent bone loss by reducing OC production (62). In addition, SIRT2 can promote OB proliferation and enhance activity through SIRT2/RUNX2 cascade regulation (63). Other studies have shown that hepatic SIRT2 gene defects can inhibit OC production and attenuate bone loss through liver-bone communication (64). FGF23 can inhibit the conversion of 25-hydroxy vitamin D to active 1,25(OH)2D3 by targeting the renal proximal tubule (65) and also affects bone mineralization and bone homeostasis by inhibiting OB function, which ultimately leads to bone destruction and increased fracture risk (66). It has been suggested that FGF23 is positively correlated with impaired bone trabecular microarchitecture, and it can be used as one of the predictors of trabecular bone loss (67). However, other clinical studies have concluded that there is no direct correlation between FGF23 and bone parameters (68). We found that genetically predicted IFN-γ, β-NGF, and SIRT2 can decrease fracture risk, whereas FGF23 promotes fracture occurrence. Studies related to the role of these CIPs in the skeletal system are controversial, and our study can provide reference evidence.

Our study has the following advantages. Firstly, this is the first MR analysis to explore the causal associations of 91 CIPs with OP and fractures. Secondly, bidirectional TSMR analysis can effectively reduce bias caused by confounding factors, environmental factors, and reverse causality. Thirdly, there are numerous but controversial studies reported on the associations of CIPs with OP and fracture and bone homeostatic imbalance, and the present study can provide insightful information for research in this area from a genetic perspective. This study also has some limitations. First, although sensitivity analyses of MR verified the validity and robustness of IVs, the possibility of residual heterogeneity remains. Second, we only utilized databases of European ancestry for the MR analyses, and the conclusions should be interpreted with caution when applied to other populations. Third, this study only explored the genetic associations between a limited number of inflammatory proteins with OP and fractures. Actually, there may be more inflammatory proteins and genetic factors, which are limited by the amount of information in the current GWAS dataset.

## 5 Conclusion

Osteoimmunology is receiving increasing attention, with CIPs playing a key bridging role between the immune and skeletal systems. We found the causal associations between multiple genetically predicted CIPs and the risk of OP and 9 types of fractures by MR analysis. The present study effectively reduces bias due to confounding factors, environmental factors, and reverse causality, and the results have favorable validity and robustness. Our study provides reliable genetic evidence for further investigation of the pathogenic mechanism of CIPs involved in bone homeostatic imbalance and finds novel potential targets for OP and fractures. With the rapid development of the post-genomic era, the integration of more up-to-date datasets to map the network of genetic associations between CIPs and OP and fractures is an interesting research direction.

## Data availability statement

The original contributions presented in the study are included in the article/**Supplementary Material**. Further inquiries can be directed to the corresponding authors.

## Ethics statement

Ethical review and approval was not required for the study on human participants in accordance with the local legislation and institutional requirements. Written informed consent from the patients/participants or patients/participants’ legal guardian/next of kin was not required to participate in this study in accordance with the national legislation and the institutional requirements.

## Author contributions

QZ: Writing – original draft, Supervision, Formal analysis, Data curation, Conceptualization. DW: Writing – original draft, Software, Investigation. RL: Writing – original draft, Project administration, Methodology. ZL: Writing – original draft, Validation. YC: Writing – original draft, Visualization, Resources. RC: Writing – review & editing, Visualization, Project administration. CZ: Writing – review & editing, Validation, Supervision. WX: Writing – review & editing, Validation, Supervision, Funding acquisition, Conceptualization.
