# Supplementary material for: Effects of circulating inflammatory proteins on osteoporosis and fractures: evidence from genetic correlation and Mendelian randomization study
Source: Front Endocrinol (Lausanne). 2024 May 1;15:1386556. doi: 10.3389/fendo.2024.1386556 (PMC11097655; doi:10.3389/fendo.2024.1386556)
Supplement: Supplementary file 1 [file DataSheet_1.pdf]

Supplementary Material

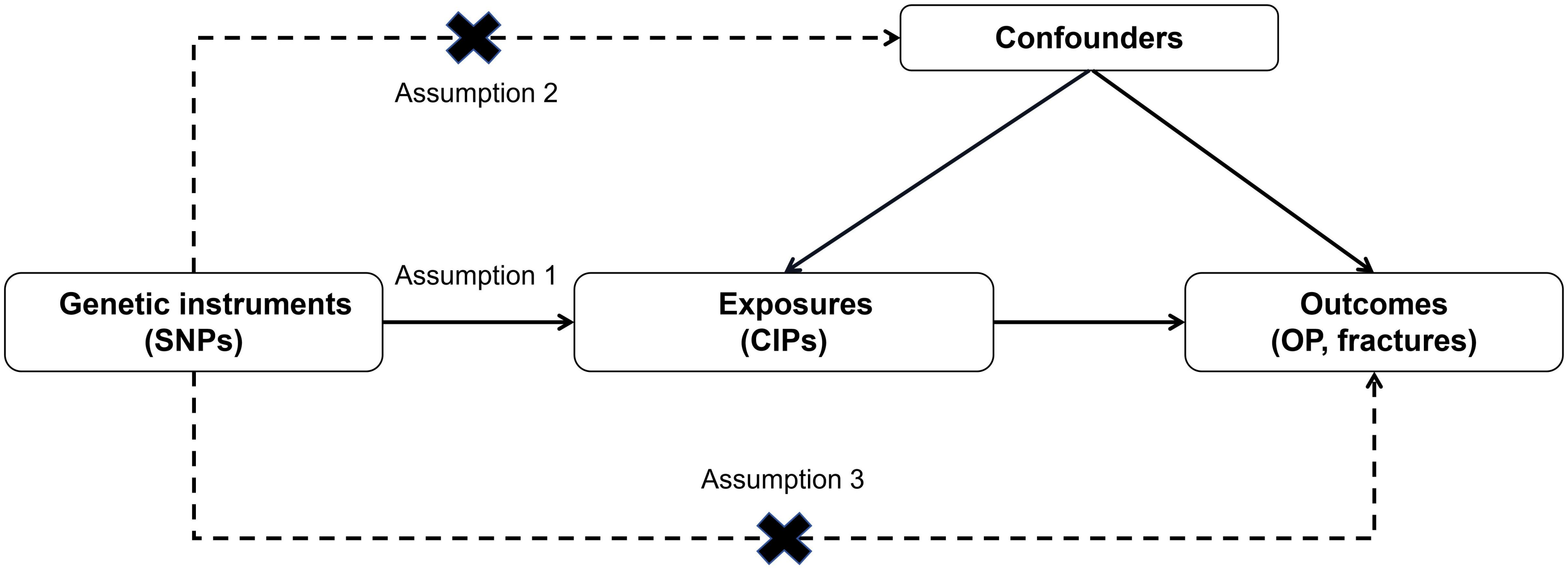

Figure S1. Assumptions in MR analysis. Three assumptions of MR are as follows: (1) Assumption 1: genetic instruments must be significantly associated with the exposure; (2) Assumption 2: genetic instruments are independent of confounders; (3) Assumption 3: genetic instruments affect the outcome only via exposure. SNP, single-nucleotide polymorphism; CIPs, circulating inflammatory proteins; OP, osteoporosis.

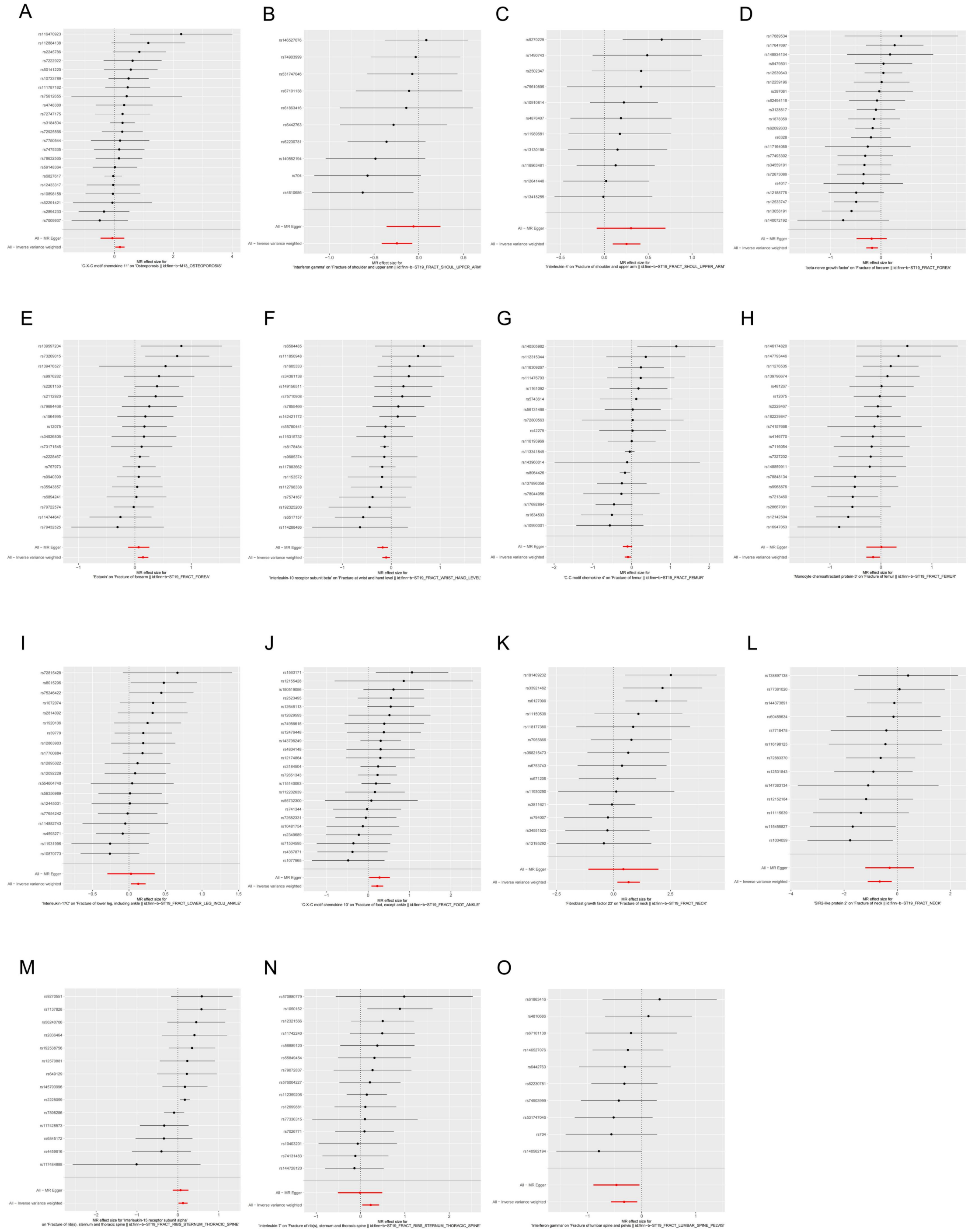

Figure S2. Forest plots of causal associations between exposures (CIPs) and outcomes (OP, fractures). (A) Forest plot between CXCL11 and OP; (B) Forest plot between IFN-γ and fracture of shoulder and upper arm; (C) Forest plot between IL-4 and fracture of shoulder and upper arm; (D) Forest plot between β-NGF and fracture of forearm; (E) Forest plot between eotaxin and fracture of forearm; (F) Forest plot between IL-10RB and fracture at wrist and hand level; (G) Forest plot between CCL4 and fracture of femur; (H) Forest plot between MCP-3 and fracture of femur; (I) Forest plot between IL-17C and fracture of lower leg (including ankle); (J) Forest plot between CXCL10 and fracture of foot (except ankle); (K) Forest plot between FGF23 and fracture of neck; (L) Forest plot between SIRT2 and fracture of neck; (M) Forest plot between IL-15RA

and fracture of rib(s), sternum and thoracic spine; (N) Forest plot between IL-7 and fracture of rib(s), sternum and thoracic spine; (O) Forest plot between IFN-γ and fracture of lumbar spine and pelvis.

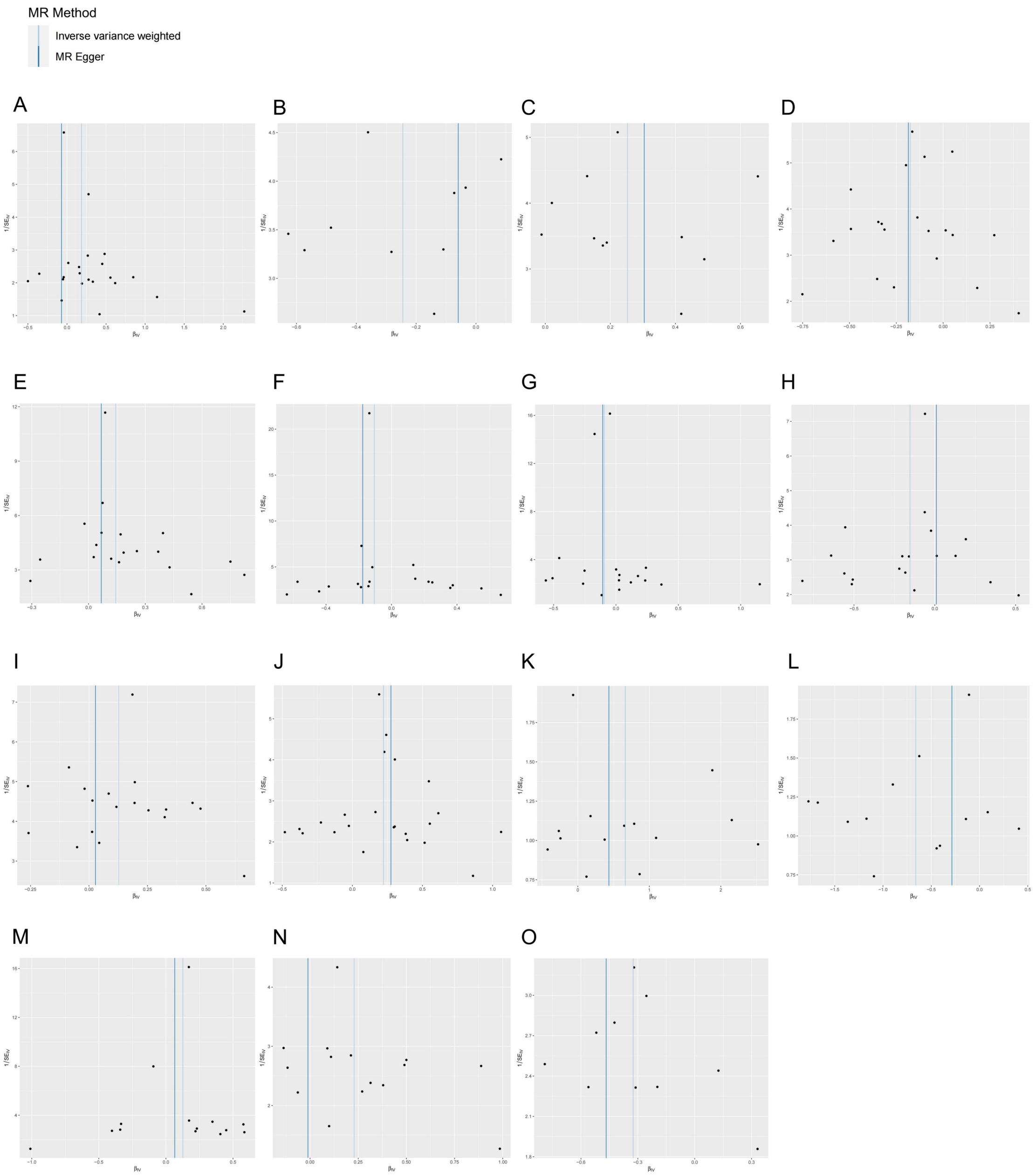

Figure S3. Funnel plots of causal associations between exposures (CIPs) and outcomes (OP, fractures). (A) Funnel plot between CXCL11 and OP; (B) Funnel plot between IFN-γ and fracture of shoulder and upper arm; (C) Funnel plot between IL-4 and fracture of shoulder and upper arm; (D) Funnel plot between β-NGF and fracture of forearm; (E) Funnel plot between eotaxin and fracture of forearm; (F) Funnel plot between IL-10RB and fracture at wrist and hand level; (G) Funnel plot between CCL4 and fracture of femur; (H) Funnel plot between MCP-3 and fracture of femur; (I) Funnel plot between IL-17C and fracture of lower leg (including ankle); (J) Funnel plot between CXCL10 and fracture of foot (except ankle); (K) Funnel plot between FGF23 and fracture of neck; (L) Funnel plot between SIRT2 and fracture of neck; (M) Funnel plot between IL-15RA and fracture of rib(s), sternum and thoracic spine; (N) Funnel plot between IL-7 and

fracture of rib(s), sternum and thoracic spine; (O) Funnel plot between IFN-γ and fracture of lumbar spine and pelvis.

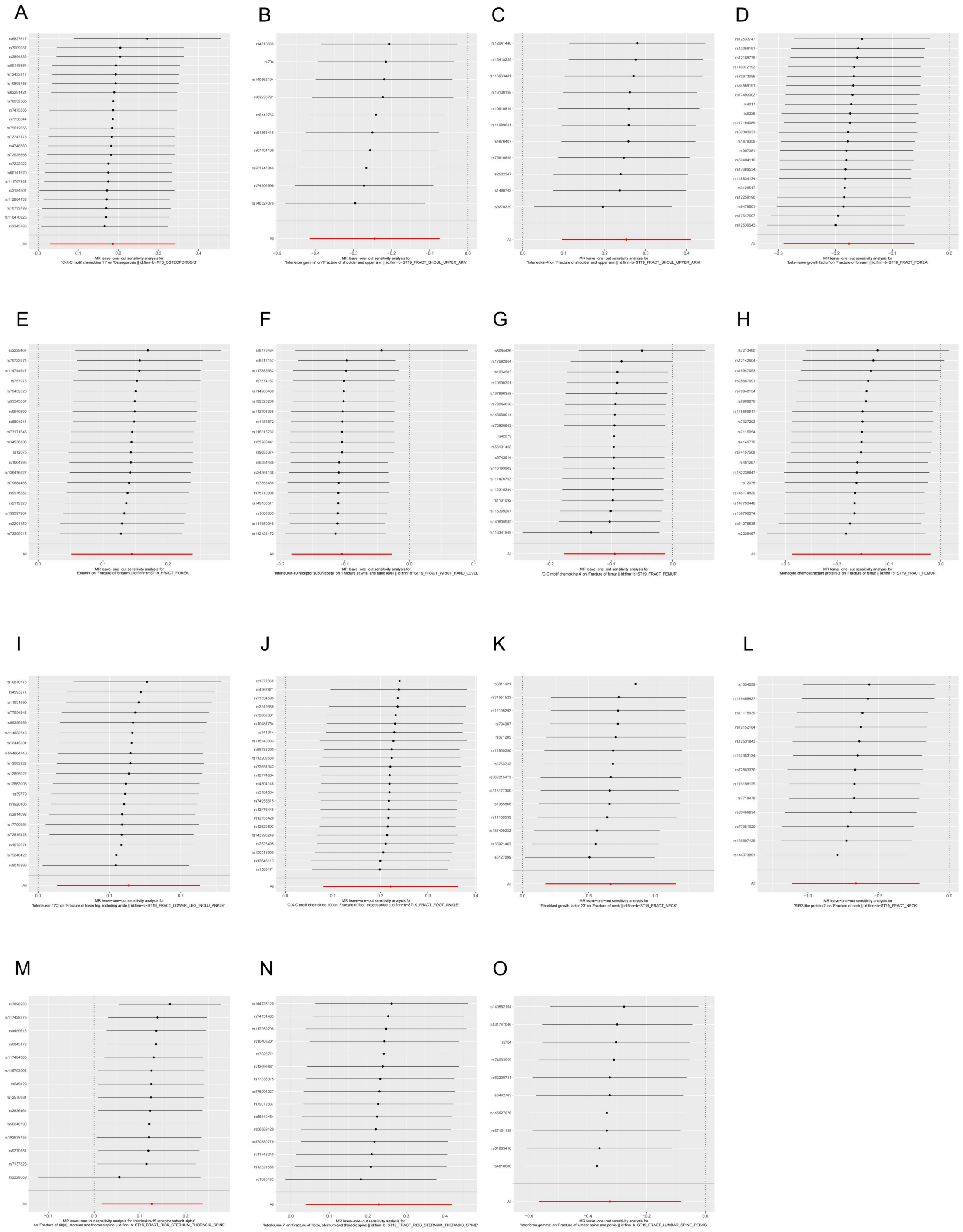

Figure S4. Leave-one-out analysis plots of causal associations between exposures (CIPs) and outcomes (OP, fractures). (A) Leave-one-out analysis plot between CXCL11 and OP; (B) Leave-one-out analysis plot between IFN-γ and fracture of shoulder and upper arm; (C) Leave-one-out analysis plot between IL-4 and fracture of shoulder and upper arm; (D) Leave-one-out analysis plot between β-NGF and fracture of forearm; (E) Leave-one-out analysis plot between eotaxin and fracture of forearm; (F) Leave-one-out analysis plot between IL-10RB and fracture at wrist and hand level; (G) Leave-one-out analysis plot between CCL4 and fracture

of femur; (H) Leave-one-out analysis plot between MCP-3 and fracture of femur; (I) Leave-one-out analysis plot between IL-17C and fracture of lower leg (including ankle); (J) Leave-one-out analysis plot between CXCL10 and fracture of foot (except ankle); (K) Leave-one-out analysis plot between FGF23 and fracture of neck; (L) Leave-one-out analysis plot between SIRT2 and fracture of neck; (M) Leave-one-out analysis plot between IL-15RA and fracture of rib(s), sternum and thoracic spine; (N) Leave-one-out analysis plot between IL-7 and fracture of rib(s), sternum and thoracic spine; (O) Leave-one-out analysis plot between IFN-γ and fracture of lumbar spine and pelvis.

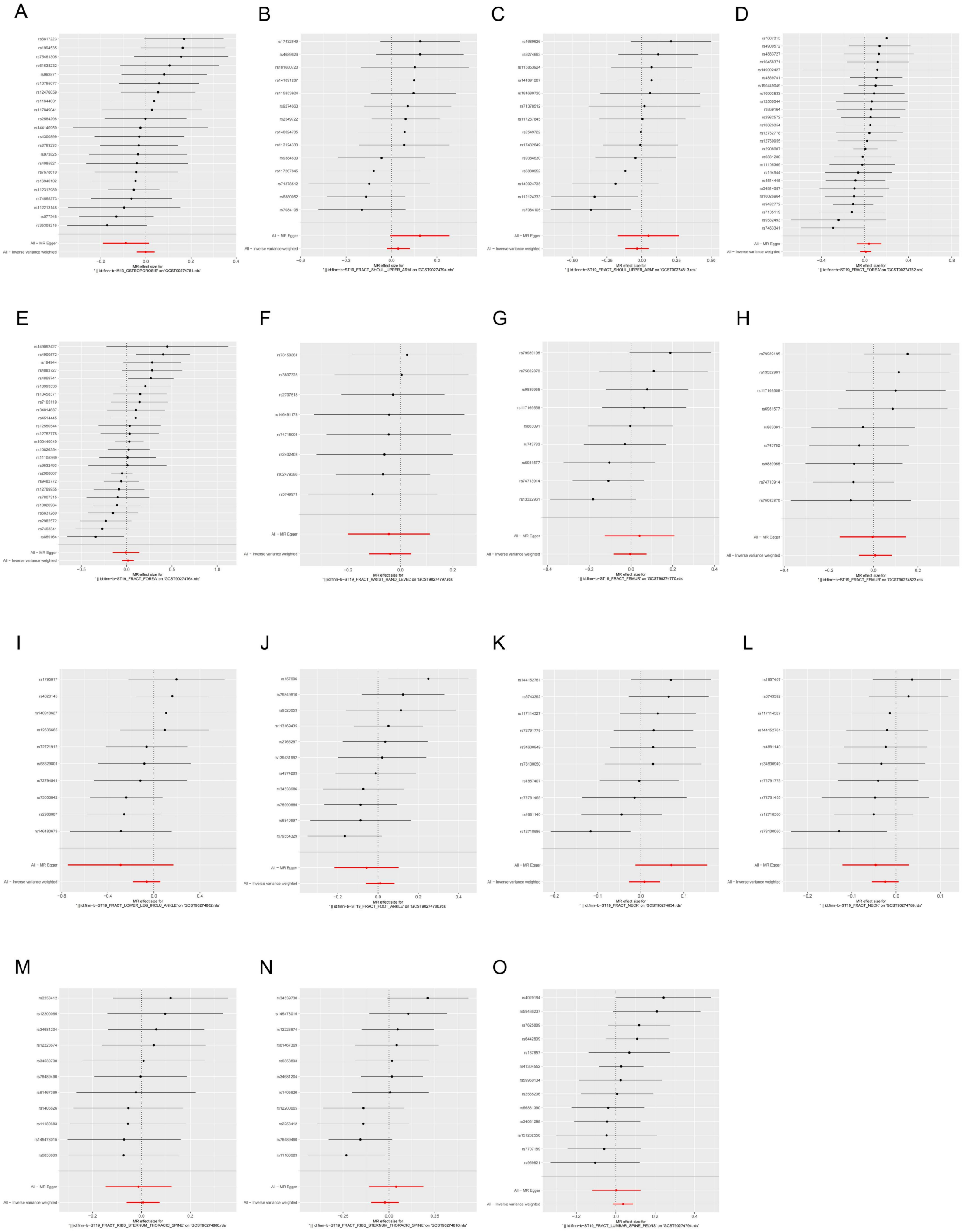

Figure S5. Forest plots of causal associations between exposures (OP, fractures) and outcomes (CIPs). (A) Forest plot between OP and CXCL11; (B) Forest plot between fracture of shoulder and upper arm and IFN-γ; (C) Forest plot between fracture of shoulder and upper arm and IL-4; (D) Forest plot between fracture of forearm and β-NGF; (E) Forest plot between fracture of forearm and eotaxin ; (F) Forest plot between fracture at wrist and hand level and IL-10RB; (G) Forest plot between fracture of femur and CCL4; (H) Forest plot between fracture of femur and MCP-3; (I) Forest plot between fracture of lower leg (including ankle) and IL-17C; (J) Forest plot between fracture of foot (except ankle) and CXCL10; (K) Forest plot between fracture of neck and FGF23; (L) Forest plot between fracture of neck and SIRT2; (M) Forest plot between fracture of rib(s), sternum and thoracic spine and IL-15RA; (N) Forest plot between fracture of rib(s), sternum and thoracic spine and IL-7; (O) Forest plot between fracture of lumbar spine and pelvis and IFN-γ.

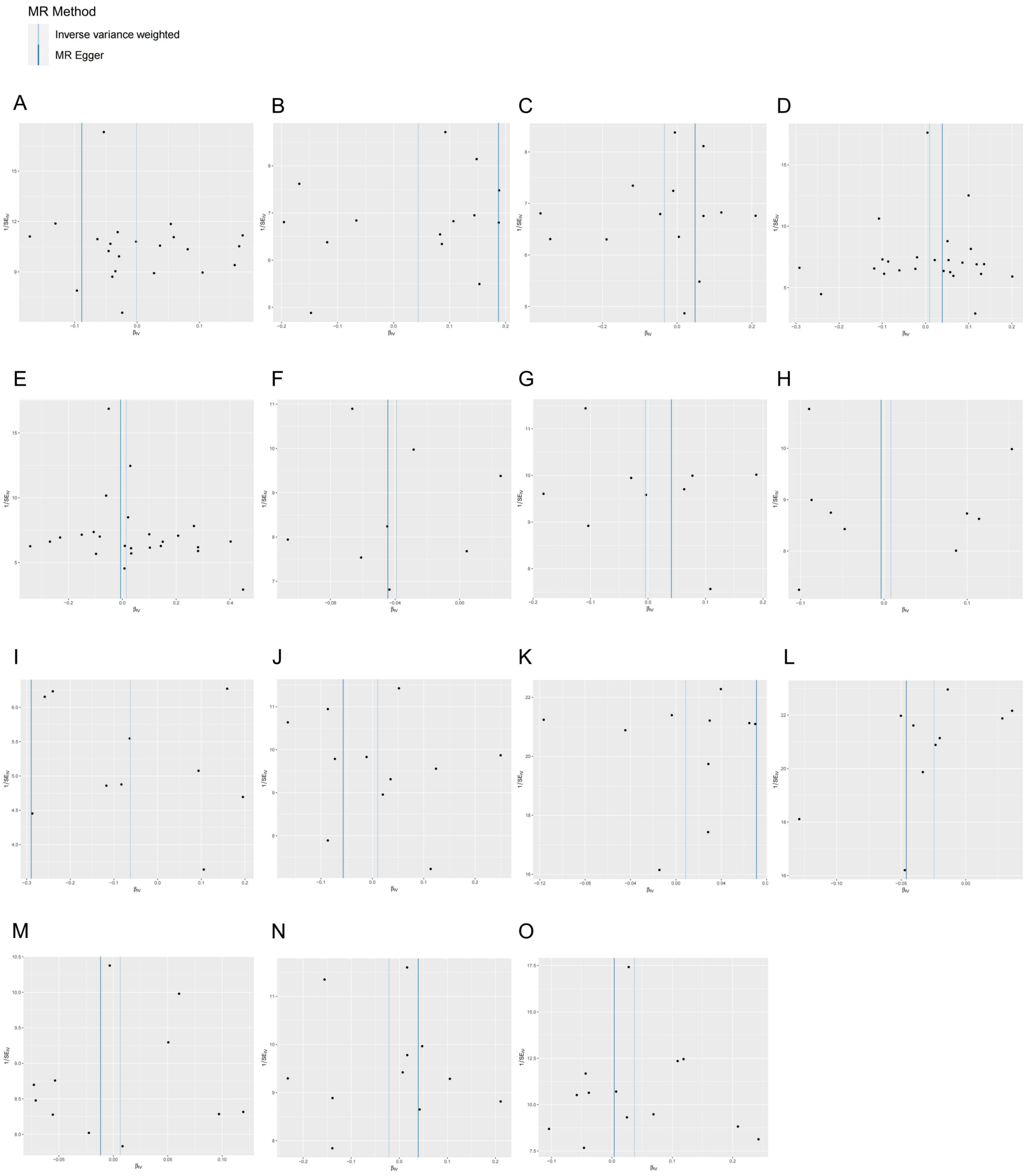

Figure S6. Funnel plots of causal associations between exposures (OP, fractures) and outcomes (CIPs). (A) Funnel plot between OP and CXCL11; (B) Funnel plot between fracture of shoulder

and upper arm and IFN-γ; (C) Funnel plot between fracture of shoulder and upper arm and IL-4; (D) Funnel plot between fracture of forearm and β-NGF; (E) Funnel plot between fracture of forearm and eotaxin ; (F) Funnel plot between fracture at wrist and hand level and IL-10RB; (G) Funnel plot between fracture of femur and CCL4; (H) Funnel plot between fracture of femur and MCP-3; (I) Funnel plot between fracture of lower leg (including ankle) and IL-17C; (J) Funnel plot between fracture of foot (except ankle) and CXCL10; (K) Funnel plot between fracture of neck and FGF23; (L) Funnel plot between fracture of neck and SIRT2; (M) Funnel plot between fracture of rib(s), sternum and thoracic spine and IL-15RA; (N) Funnel plot between fracture of rib(s), sternum and thoracic spine and IL-7; (O) Funnel plot between fracture of lumbar spine and pelvis and IFN-γ.

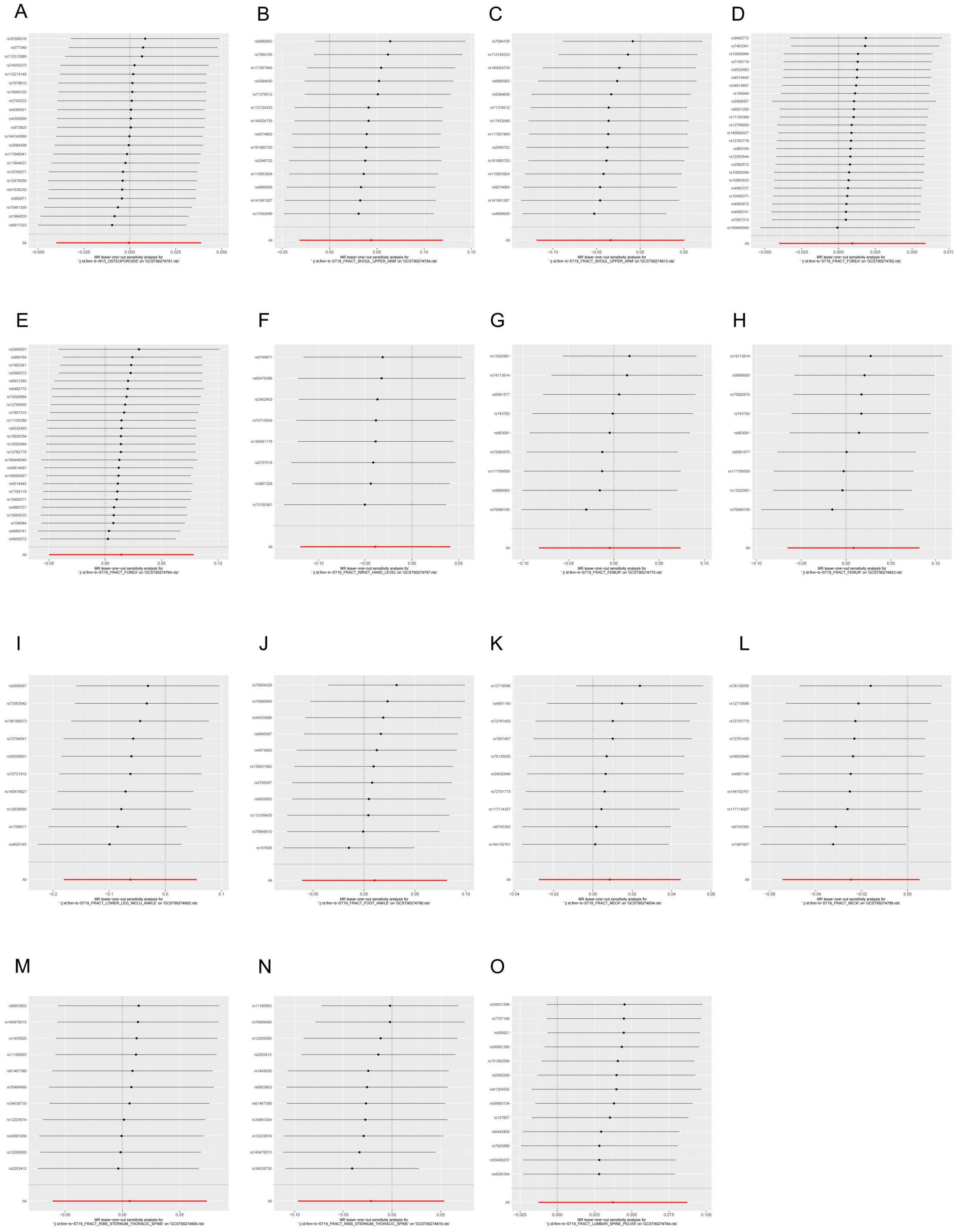

Figure S7. Leave-one-out analysis plots of causal associations between exposures (OP, fractures) and outcomes (CIPs). (A) Leave-one-out analysis plot between OP and CXCL11; (B) Leave-one-out analysis plot between fracture of shoulder and upper arm and IFN-γ; (C) Leave-one-out analysis plot between fracture of shoulder and upper arm and IL-4; (D) Leave-one-out analysis plot between fracture of forearm and β-NGF; (E) Leave-one-out analysis plot between fracture of forearm and eotaxin ; (F) Leave-one-out analysis plot between fracture at wrist and hand level and IL-10RB; (G) Leave-one-out analysis plot between fracture of femur and CCL4; (H) Leave-one-out analysis plot between fracture of femur and MCP-3; (I) Leave-one-out analysis plot between fracture of lower leg (including ankle) and IL-17C; (J) Leave-one-out analysis plot between fracture of foot (except ankle) and CXCL10; (K) Leave-one-out analysis plot between fracture of neck and FGF23; (L) Leave-one-out analysis plot between fracture of neck and SIRT2; (M) Leave-one-out analysis plot between fracture of rib(s), sternum and thoracic spine and IL-15RA; (N) Leave-one-out analysis plot between fracture of rib(s), sternum and thoracic spine and IL-7; (O) Leave-one-out analysis plot between fracture of lumbar spine and pelvis and IFN-γ.
